# Does Living in a Protected Area Reduce Resource Use and Promote Life Satisfaction? Survey Results from and Around Three Regional Nature Parks in Switzerland

**DOI:** 10.1007/s11205-023-03164-z

**Published:** 2023-07-07

**Authors:** Thea Xenia Wiesli, Wojtek Przepiorka

**Affiliations:** 1 Centre for Development and Environment, University of Bern, Bern, Switzerland; 2 Department of Sociology, Utrecht University, Utrecht, Netherlands

**Keywords:** Protected areas, Nature parks, Life satisfaction, Resource use, Ecological footprint

## Abstract

Regional nature parks in Switzerland are, for the most part, protected areas that aim to promote sustainable development and residents’ well-being. In recent years, research on regional nature parks and comparable protected areas has focused on questions regarding local populations’ acceptance of such areas, their governance, and their economic effects. However, we know surprisingly little about the impact of protected areas on environmental resource use and life satisfaction, two essential ingredients of sustainable regional development. In this study, we survey people living in and around three regional nature parks in Switzerland on their resource use and life satisfaction (gross sample *n* = 3358). We propose a novel measurement of resource use based on vignettes describing different lifestyles, which we validate against the carbon footprint obtained for a subsample of our respondents. With these indicators, using multiple regression analyses, we test several hypotheses derived from the literature on the relationship between resource use and life satisfaction in and around protected areas. Contrary to our expectations, we do not find differences in resource use or life satisfaction, or the relationship between resource use and life satisfaction, across park and non-park regions. We discuss potential explanations for our findings and their implications for nature park authorities and future study designs.

**Supplementary Information:**

The online version contains supplementary material available at 10.1007/s11205-023-03164-z.

## Introduction

Protected areas cover 26.4% of Europe (EEA, 2022). How do these protected areas affect people’s use of natural resources and their life satisfaction? We address this question with an empirical case study that focuses on a category of protected areas in Switzerland: called “Regional Nature Parks” (RNPs). In Switzerland, regions with high natural and landscape values and a traditional, cultural, scenic, or historical character can be nominated to be labeled as RNPs. The Swiss government’s aim in establishing such areas is to promote sustainable development and to contribute to people’s well-being (Federal Office for the Environment, 2019). Existing RNPs comprise a large number of rural communities and sometimes extend across several cantons (i.e. federal states). RNPs are of different sizes but include those that are over 100 km^2^ in area, and are sometimes fairly populated (Federal Office for the Environment, 2019).

If an area is nominated to become an RNP, the people living in the designated area vote on whether their region should receive the RNP label. If their consent is given, a park body is established that consists of experts in biodiversity, forestry, environmental education, renewable energies, scientific cooperation, etc. (Federal Office for the Environment, 2019). Together with representatives of the population and other interest groups, the park body develops a 10-year charter, which serves as a planning instrument, and is responsible for implementing the charter’s objectives in cooperation with the municipalities included within the RNP’s area. The general objectives of these charters are to promote people’s environmental awareness, advance the federal biodiversity strategy, improve the quality of the landscape, promote local production chains and cycles, and promote sustainable tourism (Federal Office for the Environment, 2019). The park management body receives financial support from each of the three levels of government (municipalities, cantons, federal government). In light of these investments, the question arises as to how RNP status contributes to sustainable regional development.

Previous studies suggest that environmental awareness and public infrastructure supporting pro-environmental behavior (e.g. hiking and bicycle trails, public transport) can reduce individuals’ use of natural resources (Bruderer Enzler & Diekmann, 2019; Kennedy et al., 2015; Moser & Kleinhückelkotten, 2018; Schneidewind, 2013). Moreover, research shows that high levels of landscape quality and biodiversity are positively related to people’s well-being (e.g. Bieling et al., 2014; Bignante, 2015; Carrus et al., 2015; Mossabir et al., 2021). For example, Bonet-García et al. (2015) found that the inhabitants of a large protected area in southern Spain rated their personal well-being higher than respondents in surrounding communities. According to the authors, this was a result of the efforts of the Andalusian regional government, which had made attempts to increase the well-being of the population by means of establishing protected areas since 1989. These efforts included, for example, promoting sustainable farming, public infrastructure, nature-based tourism, and forest management (Bonet-García et al., 2015). However, previous studies have also found a positive relationship between the degree of resource use and well-being (e.g. O’Neill et al., 2018), which is detrimental to sustainable development.

To our knowledge, no previous study has investigated the potential impact of RNPs on the relationship between people’s resource use and their well-being. This is a notable omission, given the multiple goals of protected areas to reduce resource use and increase well-being, on the one hand, and the common finding that resource use and well-being are positively related, on the other. We therefore ask the following two questions: (1) Is resource use lower and well-being higher in RNPs than in comparable rural regions without park status? (2) Is the positive relationship between resource use and well-being weaker in RNPs than in comparable rural regions without park status?

Addressing similar questions, Vita et al. (2020) compared resource use and socio-economic variables that influenced well-being between members and non-members of environmental grassroots initiatives. They found that membership was associated with a lower carbon footprint and higher well-being. In the study at hand, we survey individuals living in three Swiss RNPs and comparable non-park regions on their resource use and well-being in terms of *life satisfaction*. In line with Vita et al. (2020), we conceive of life satisfaction as “the cognitive component of subjective well-being”(2020, *p*. 4) (see also Brulé, 2022; Ortiz-Ospina & Roser, 2013); we define life satisfaction as a person’s current subjective attitude toward their life in general. Moreover, we define resource use as any human activity that triggers the emission of greenhouse gases (e.g. CO_2_) (Brulé, 2022; Vita et al., 2020; Wackernagel, 1994). In our study, we measure resource use by means of a proxy that captures individual lifestyles in terms of the consumption of food, and use of different modes of shelter and mobility—spheres of life that are most strongly associated with resource use (Jungbluth et al., 2011). Our measure does not account for resource use in the production of goods and services.

The remainder of our paper is structured as follows. We first outline our theoretical argument and state our hypotheses. We then describe our measurement and data analysis strategy, followed by presenting our results. Finally, we discuss our findings in light of previous research and conclude.

## Theory and Hypotheses

### The Relation Between Resource Use and Life Satisfaction

The relationship between income, which is highly related to resource use and CO_2_ emissions (Baiocchi et al., 2010; Bruderer Enzler & Diekmann, 2019; Büchs & Schnepf, 2013; Notter et al., 2013), and well-being has been repeatedly explored. The Easterlin paradox, for example—one of the early findings in this area—suggests a positive influence of income on life satisfaction up to a specific point; when income exceeds this point, life satisfaction no longer increases (Easterlin, 1974). In a similar vein, the “treadmill of production” theory (Schnaiberg et al., 2002) and the threshold hypothesis on “economic growth and quality of life” (Max-Neef, 1995) suggest that a society’s economic growth benefits, respectively, hedonic happiness and life satisfaction, but only up to a certain point. The decline at high levels of economic development is explained by high levels of consumption, which harm nature and the environment. Thus, beyond a certain threshold, “if there is more economic growth, quality of life may begin to deteriorate” (Max-Neef, 1995, p. 117).

These theories are supported by empirical evidence at the country level. Within their sample of 150 nations, O’Neill et al. (2018) did not find a single nation capable of meeting the basic needs of its citizens without overusing natural resources. One of the nations investigated was Switzerland (O’Neill et al., 2018). Average life satisfaction in Switzerland is high (OECD, 2020). At the same time, with an average of 13.2 tons of carbon emissions per capita, Switzerland far exceeds the planetary boundary benchmark of 1.6 tons per capita (O’Neill et al., 2018; Swiss Federal Statistics, 2006). However, in their analysis of 120 countries with growing per capita consumption, Fanning and O’Neill (2019) did not find significant changes in happiness (as a dimension of well-being). They even found that happiness slightly decreased above a certain level of income. Apergis and Majeed (2021) reported results from a study of 95 countries showing that greenhouse gas emissions reduce cross-national happiness levels, although economic affluence enhances these levels.

Rational choice theory argues that individuals act in a way that maximizes their utility by, for example, consuming goods and services that benefit them (e.g. Jackson, 2005; Varian, 1992). Relatedly, the capability approach suggests that goods also enable people to pursue certain goals (Nussbaum & Sen, 1993). Hence, capabilities are essential prerequisites for achieving a satisfactory life (Nussbaum & Sen, 1993). Books, computer equipment, electricity, and cars, for example, are goods that enable people to be mobile and to obtain an education, which is known to increase people’s life satisfaction. In addition, Veblen’s “theory of the leisure class” links consumption and excessive lifestyles to a prestige-generating function, which mainly serves increasing people’s social status (1973). Veblen (1973) thus suggests that the effects of consumption go beyond the fulfillment of basic needs. In summary, these theories underline that consumption plays a major role in people’s ability to achieve life satisfaction (although life satisfaction does not depend on consumption alone). At the same time, individuals’ consumption and use of economic goods trigger a large proportion of carbon emissions (Jungbluth et al., 2011).

To our knowledge, there is no empirical evidence for the relationship between individuals’ resource use and life satisfaction in Switzerland. However, several empirical results from other countries corroborate our expectation of a positive relationship between the two constructs. For example, based on an analysis of 14,960 households in China, Wang et al. (2015) provided evidence for a positive relationship between consumption expenditure and life satisfaction. However, in their analysis, the relationship between consumption expenditure and life satisfaction varies in strength depending on the consumption category. Wang et al. (2015) concluded that what money is spent on has a substantial bearing on life satisfaction. In line with this conclusion, Lenzen and Cummins (2013) showed that among different areas of household consumption that contribute to the carbon footprint, car ownership is positively related to well-being (see also Brulé et al. 2020). These theoretical considerations, along with the empirical evidence, lead us to our first hypothesis:

#### Hypothesis 1a

People’s resource use is positively related with their life satisfaction.

O’Neill et al.’s study of 150 nations (2018) not only found a positive relationship between the use of environmental resources and life satisfaction, but they also found that the more environmental resources are used, the slower the rate of the increase in life satisfaction. We have no reason to assume that this will be different for individuals as compared to countries. Since income and carbon footprint are linked (Baiocchi et al., 2010; Bruderer Enzler & Diekmann, 2019; Büchs & Schnepf, 2013; Notter et al., 2013), we assume that satisfaction also increases with resource use at a decreasing rate at the individual level. Due to the diminishing marginal utility of consumption, increases in consumption will affect life satisfaction to a lesser extent at high levels of consumption than at low levels. This leads us to our second hypothesis:

#### Hypothesis 1b

People's resource use increases their life satisfaction but it does so at a declining rate: the more resources they use, the slower the increase in life satisfaction.

### Resource Use and Life Satisfaction in and Around RNPs

Given the challenges posed by climate change, RNPs in Switzerland can be considered as model regions for sustainable development (Hammer et al., 2016; UNESCO, 2017). Pilot projects in RNPs are used to test new sustainable infrastructure (e.g. bicycle roads), with the results of these projects then used to guide the expansion of sustainable infrastructure throughout Switzerland (Hammer et al., 2016). Evidence that RNPs do more than non-RNP areas to promote sustainable development and environmental education, which in turn can influence ecological behavior (Bruderer Enzler & Diekmann, 2019; Kennedy et al., 2015; Moser & Kleinhückelkotten, 2018), leads us to conjecture that park inhabitants use fewer environmental resources than people living in regions where these efforts are not made.

Spatial factors (Brereton et al., 2008), climate, and air pollution (Cuñado & De Gracia, 2013) are also significant determinants of well-being. Evidence indicates that infrastructure can be designed in accordance to have a positive influence on well-being (Brereton et al., 2008; Sarmiento et al., 2022), and significant differences by region have been observed in this regard (Sarmiento et al., 2022). According to several studies, high-quality landscapes and ecosystems contribute to greater well-being in terms of mental and physical health (Abraham et al., 2010; Bieling et al., 2014; Bignante, 2015; Carrus et al., 2015; Skärbäck, 2007; Summers et al., 2012). Moreover, in line with the aims of the park label, the promotion of sustainable local economies could prevent the aging of society in rural areas, due to the phenomenon of rural exodus. Economic development also fulfills basic needs and can thus—at least up to a certain level—contribute to life satisfaction (Max-Neef, 1995). In addition, regions are nominated for the RNP label because they have a special cultural heritage, which can induce a sense of identity. For example, the participation of local actors in park management activities can be expected to strengthen inhabitants’ regional identity (Federal Office for the Environment 2019). Both cultural heritage (Hammer et al., 2011) and identity (Lengen, 2016) have been shown to contribute to people’s life satisfaction. On this basis, we anticipate that the life satisfaction of park inhabitants will be higher than that of people living in comparable non-park regions. This leads us to our next hypothesis:

#### Hypothesis 2a

People living in RNPs exhibit lower use of resources and higher life satisfaction than people living in comparable non-park regions.

Based on the current state of research, we assume that certain factors moderate (i.e. affect the strength of) the relationship between individuals’ resource use and their life satisfaction. Verhofstadt et al. (2016) suggest that an environmentally friendly diet and not using electricity for heating simultaneously decrease individuals’ resource use and increase their life satisfaction. In addition, empirical studies (O’Neill et al., 2018) and theoretical work (Schneidewind, 2013) suggest that infrastructure helps individuals adopt behaviors that reduce resource use. We expect that these factors not only affect resource use and life satisfaction directly but can also act as moderators of the relationship between the two constructs. The goals of RNPs include promoting local seasonal products (e.g. through marketing and development of product labels), renewable energy (e.g. through cooperation with municipalities, energy forums and providers, scientists, and other experts in park management), landscape and nature (e.g. through voluntary work, co-work with agriculture and forestry organizations, nature excursions, and nature conservation zones), and footpaths and cycle routes (e.g. through the initiation and maintenance of co-work with municipalities and forestry organizations). We thus expect these efforts to affect people’s lives in RNPs. Based on these arguments, we formulate our next hypothesis:

#### Hypothesis 2b

The positive relationship between resource use and life satisfaction will be weaker for people living in RNPs than for people living in comparable, non-park regions.

### RNP Age as a Moderator of the Relation Between Resource Use and Life Satisfaction

The study areas in our sample are three Swiss RNPs: Gantrisch Nature Park (GNP), Jurapark Aargau (JPA), and UNESCO Biosphere Entlebuch (UBE). The UBE is the oldest of the three RNPs. It received the “UNESCO Biosphere Reserve” label in 2001, became an RNP in 2008, and has been pursuing activities since 1998. The GNP and the JPA were both established in 2012. The UBE is thus 14 years older than the other two RNPs. Accordingly, we expect the UBE to exhibit stronger effects on inhabitants’ resource use and life satisfaction than the GNP and the JPA. In accordance with the argument leading up to hypotheses 2a and 2b, we state our last two hypotheses:

#### Hypothesis 3a

People living in the UBE exhibit lower resource use and higher life satisfaction than people living in the GNP and the JPA.

#### Hypothesis 3b

The positive relationship between resource use and life satisfaction will be weaker for people living in the UBE than for people living in the GNP and the JPA.

## Materials and Methods

We conducted an analysis of survey data to test our hypotheses. The survey data were collected by means of a postal and online survey in the three RNPs and in the surrounding control regions in 2019. The following sections describe the study areas, the data collection procedure, the data, and the analyses we conducted.

### Study Areas

**Fig. 1:**
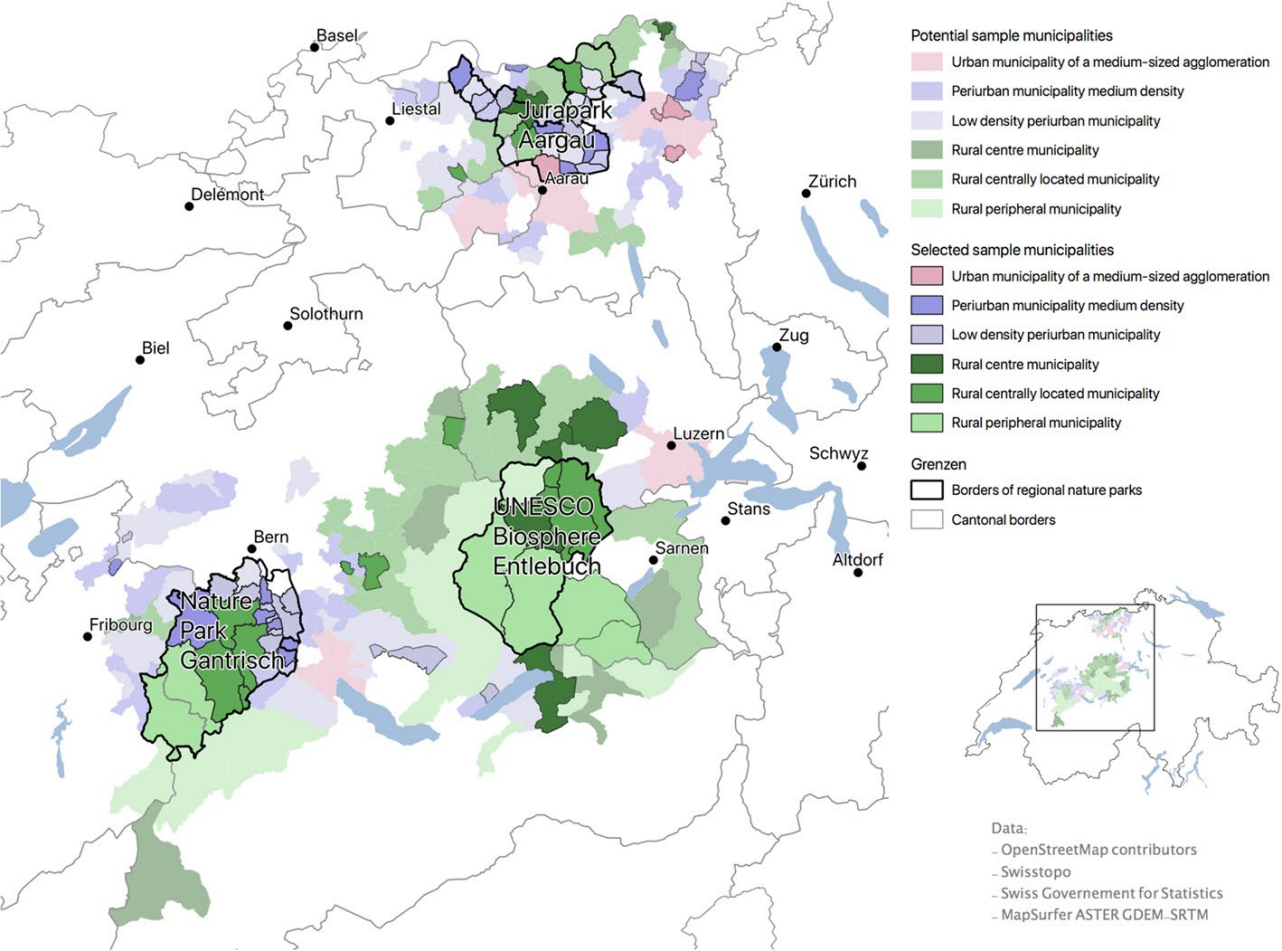
Location of the RNPs and the control group (light colors). Source: Open Street Map Contributors, Swisstopo, ESRI. Map: Anon

The three RNPs are the GNP, JPA, and the UBE. UNESCO biosphere reserves in Switzerland are subsumed under the RNP label. The three RNPs are comparable as they are located at the edge of the Swiss Plateau (see Fig. 1), are easily accessible from densely populated conurbations, and have a high population density compared to smaller alpine RNPs (e.g. 167.63 people per square kilometer in JPA) (Wiesli et al., 2022). These are typical features of RNPs in Europe. Accordingly, this selection of study areas makes it likely that the results of this study can be generalized to other Swiss and European parks.

Another reason to choose these three RNPs was their difference in age. Since we hypothesized that the age of an RNP has a moderating effect on the relationship between resource use and life satisfaction (see Hypotheses 3a and 3b), we chose to include the oldest existing RNP in Switzerland, the UBE. However, with 17,600 inhabitants, the UBE also has the smallest population of the three RNPs (Wiesli et al., 2022). It covers an area of 394 km^2^, which is less than the GNP and more than the JPA. According to the Swiss government’s typology of municipalities, the seven municipalities of the UBE are central rural and peripheral rural municipalities (Federal Statistical Office, 2012). The UBE thus has a decidedly rural character within the Swiss context. However, the UBE is located near the city of Luzern.

With 46,500 inhabitants, the GNP has the largest population of the three RNPs (Wiesli et al., 2022). Its 20 municipalities include peri-urban municipalities with medium and low population densities, as well as central rural and peripheral rural municipalities (Federal Statistical Office, 2012). The GNP is located near the city of Bern.

The JPA covers an area of 245 km^2^ and has a population of 40,400 (Wiesli et al., 2022). Its 28 municipalities include peri-urban municipalities of medium density, central rural municipalities, and medium-sized urban municipalities (Federal Statistical Office, 2012). In contrast to the other two RNPs, the JPA is more strongly characterized by urban agglomeration. The JPA is located near Zurich, the most highly populated city in Switzerland.

The control group consists of people living in municipalities around the three RNPs (see Fig. 1). Their data serve to show differences between park residents and non-park residents and to verify the influence of the RNP status on resource use and life satisfaction. To ensure comparability between the two groups, the non-park municipalities were selected so that the municipality types, the cantons, and the language spoken (German) matched those within the RNPs. Apart from these criteria, the control municipalities were selected randomly. Their degrees of urbanization and population densities are similar to those of the park municipalities, according to the typology of the Swiss Federal Statistical Office (2012). All of the sampled municipalities are shown in Fig. 1.

### Sampling

In the GNP, the JPA, and the control regions, we first sampled the municipalities and then drew a random sample of the adult population (Wiesli et al., 2022). In the UBE, due to the small number of municipalities, we selected all municipalities. In line with the Swiss municipality typology of 2012 (Federal Statistical Office, 2012), we grouped the municipalities according to their degree of urbanization and their population density (Wiesli et al., 2022). Stratified sampling was then applied to each municipality type, meaning that the sample size was proportional to the total population of the relevant municipality type.

The study was described to respondents living in the RNPs as investigating the quality of life in the park, and to respondents living in the control regions as investigating the quality of life in the given region (Wiesli et al., 2022). The ecological topic was not mentioned in the postal letter or in the online project description, in order to avoid the inclusion of a disproportionate number of people with an above-average interest in environmental topics. One reminder was sent. The resulting response rate was 25% (*n* = 3358) (Wiesli et al., 2022). The returned questionnaires covered an average of 3% of the population in the three parks.

The mean age of the sample was 50.8 years in the RNPs (*n* = 2409) and 51.4 years in the control regions (*n* = 949) (Wiesli et al., 2022) (see Table 2). The majority of participants were female, both in the RNPs (53.04%) and in the control regions (53.6%). About one-quarter of participants in the RNPs (24.4%) and control regions (25.8%) were housewives or househusbands. 43.1% of the park sample and 42.9% of the control group sample had completed an apprenticeship as their highest level of education. Moreover, 16.6% of the RNP sample and 17.2% of the control group sample worked in the service sector, and 12% of the RNP sample and 11.2% of the control group sample worked in education or the social sector. Finally, in the park sample, 73.05% were employed and 6.55% were retired, while in the control group sample, 72.3% were employed and 7.2% were retired.

Official statistics on gender and age in the park municipalities show that our sample is comparable to the park population in these respects (see Table 2) (Federal Statistical Office, 2016). Data on education and employment were not available at the municipality level, so we can only compare our sample to the whole of Switzerland. Our sample resembles the Swiss population in both regards: 40% of people living in Switzerland have an apprenticeship as their highest qualification, and 68.1% are employed (Federal Statistical Office 2016).

### Life Satisfaction

The main outcome variable was respondents’ general life satisfaction (“How satisfied are you with your life in general?”), measured on a scale from 0 (= “not at all satisfied”) to 10 (= “fully satisfied”). To validate this variable, we created a global index of satisfaction including 21 items (*M* = 7.98, Cronbach’s *α* = 0.837, *n* = 3358) relating to satisfaction with specific areas of life (e.g. “How satisfied are you with your job?”), measured on the same 11-point scale as general life satisfaction. We calculated the relationship between the general life satisfaction variable and the global index by using Spearman correlation and ordinary least squares (OLS) regression (see Table 1). Since the correlation coefficient (*r* = 0.505, *p* < 0.001) and the coefficients from simple (*b* = 0.697, *p* < 0.001) and multiple OLS regressions (*b* = 0.674, *p* < 0.001) were positive and statistically significant, we conclude that our single variable of general life satisfaction is a valid measure of life satisfaction. The control variables included in the OLS regression are known to influence individuals’ life satisfaction (e.g. Frey & Stutzer, 2018). In our models, age, gender, and household income confirm other studies and influence life satisfaction significantly (see Table 1). Variables of education (years of education) and residency status (whether or not individuals are in possession of a Swiss passport) do not have a statistically significant influence on our findings.

**Table 1:** Correlation using Spearman and OLS regression to validate the resource use indicator with the carbon footprint

	Satisfaction					Resource use				
	a	b		c		d	e		f	
	*r*	Coef	SE	Coef	SE	*r*	Coef	SE	Coef	SE
Satisfaction index (21 variables)	0.505***	0.697***	0.020	0.674***	0.333					
Carbon footprint [kg CO_2_ eq.] (log.)						0.177***	1.078***	0.168	1.242***	0.188
Age (years)				0.006**	0.002				− 0.017*	0.008
Gender (female = 1)				0.122*	0.059				− 0.733***	0.209
Household income (in 10 CHF)				0.002*	0.001				− 0.012***	0.003
Education (years)				0.002	0.015				− 0.108*	0.052
Swiss passport (yes = 1)				− 0.214	0.120					
Parent (yes = 1)									− 0.062	0.244
Constant		2.785	0.165	2.642***	0.340		1.156	1.465	3.280	1.767
Number of observations	3303	3303		1189		1358	1358		1202	
Adjusted *R*^2^		0.260		0.283			0.029		0.061	

The table lists Spearman correlation in Model a, a simple OLS regression in Model b, and a multiple OLS regression in Model c, with general life satisfaction as the outcome variable and a global index of satisfaction composed of 21 items of satisfaction with several life areas as income variable (***p < 0.001, **p < 0.01, *p < 0.05). Model d presents a Spearman correlation, Model e a simple OLS regression, and Model f a multiple OLS regression with a proxy of resource use, measured by lifestyle descriptions as the outcome variable and the carbon footprint as the income variable

We furthermore conducted an exploratory factor analysis (EFA) with the 21 items used to create the global index. These items loaded on two factors, one measuring satisfaction with infrastructure (e.g. public transport) and another measuring satisfaction with the respondent’s financial situation (e.g. cost of housing). We conducted additional analyses with these two factors, instead of general life satisfaction, as the outcome variable, in order to compare and validate our main results. These additional analyses are reported in Appendix A.

### Resource Use

The main explanatory variable was an indicator of resource use (*n* = 2782). This variable was constructed on the basis of three vignettes that were presented to every respondent. These vignettes contained short lifestyle descriptions focusing on the most resource-intensive behaviors at the individual and household levels in countries such as Switzerland. According to the carbon footprint calculation of the World Wide Fund for Nature (WWF, 2019), these behaviors relate to mobility, housing, and nutrition. For example, vignette C described a lifestyle with high resource use and read as follows:

*Person C lives in a spacious house in the countryside and appreciates having a lot of space. In terms of energy sources for the house, the person mainly uses electricity from mixed sources and gas for heating. […] He/she enjoys traveling to warm countries and flies to South Africa once a year. His/her goal is to see New Zealand soon […].*

Vignettes A and B described lifestyles with low and medium resource use, respectively. The survey participants were asked to indicate, on an 11-point scale from 0 (= *“does not apply”*) to 10 (= *“applies”*), to what degree each of the three lifestyles applied to them. We excluded 142 cases in which respondents scored less than four on all three vignettes in total, as these did not provide sufficient information on respondents’ resource use. We created the indicator of resource use by subtracting the vignette A item (low resource use) from the vignette C item (high resource use) and adding 11 so that the resulting variable of resource use ranged from 1 (= lowest resource use) to 21 (= highest resource use).

Furthermore, we calculated a shortened version of the carbon footprint for a subsample of our survey participants (*n* = 1526). This variable was calculated for each respondent based on a procedure developed for WWF (2019) by Jungbluth and Meili (2017). In contrast to the calculation by WWF, we only included the consumption categories of mobility, shelter, and food (Jungbluth et al., 2011).^1^ To obtain the corresponding data, participants were asked to provide information about their behavior relating to these three categories (e.g. “Do you use a car or motorbike in your private life?”, “How big is your apartment/house?”, “Which of the statements below describes your diet? Meat or fish daily, …weekly”, etc.). The carbon footprint of nutrition was calculated as a function of the consumption of meat and dairy products. The carbon footprint of mobility was calculated as a function of kilometers traveled by private transport (e.g. car), by public transport (e.g. train), and by air. The carbon footprint of shelter was calculated as a function of the respondent’s living space, the type of heating, and the number of people in the household. Based on the combination of these categories, the estimated carbon footprint expressed in annual kg of CO_2_ equivalents was calculated for each respondent in this subsample.

However, since the carbon footprint variable contained significantly fewer cases than the full sample, we only used it to validate our vignette-based indicator of resource use. We calculated the relationship between the log-transformed carbon footprint variable and the indicator of resource use by using Spearman correlation and OLS regression (Table 1). Since the correlation coefficient (*r* = 0.177, *p* < 0.001) and the coefficients from simple (*b* = 1.078, *p* < 0.001) and multiple OLS regressions (*b* = 1.241, *p* < 0.001) were positive and statistically significant, we conclude that our vignette-based indicator is a valid measure of resource use.

The control variables included in the OLS regression are known to influence individuals’ resource use (e.g. Bruderer Enzler & Diekmann, 2019; Diekmann & Preisendörfer, 2001). Age, gender, household income, and years of education influence resource use significantly. The variable indicating whether the respondent had children does not have any significant influence.

### Control Variables

When testing our hypotheses by means of multiple regression, we controlled for age, gender, household size and income, education, and whether the respondent was a Swiss citizen (Table 2). According to Frey and Stutzer (2018), on average, women are slightly more satisfied with their lives than men, younger and older people are more satisfied with their lives than middle-aged people (suggesting a u-shaped relation between age and life satisfaction), nationals are more satisfied with their lives than foreigners, and people living in collective households are more satisfied with their lives than people living in single households. The influence of income on life satisfaction is controversial. Life satisfaction does not increase gradually and infinitely with rising income (Frey & Stutzer, 2018). Nevertheless, Frey and Stutzer summarized that people with high incomes reported higher satisfaction than people with low incomes. In our sample, income was assessed as gross household income, in income classes (e.g. CHF 4001–6000 = class 3). This ordinal variable was recoded into a continuous variable using category means and divided by 10 to simplify interpretation in the OLS regression models.

**Table 2:** Descriptive statistics of the variables included in the regression models

	Full sample			Park sample			Park population			Control group sample		
	*M*	*SD*	*n*	*M*	*SD*	*n*	*M*	*SD*	*n*	*M*	*SD*	*n*
Satisfaction (0–10)	8.36	1.34	3328	8.38	1.32	2387	–	–	–	8.31	1.39	941
Resource use (1–21)	10.44	3.58	2782	10.49	3.60	2001	–	–	–	10.31	3.53	781
Age (years)	51.41	17.60	3358	50.85	17.55	2409	49.6	7.2	78,939	51.41	17.75	949
Gender (female = 1)	0.53	0.50	3347	0.53	0.50	2400	0.50	–	78,939	0.54	0.50	947
Single household (no = 1)	0.86	0.34	3308	0.87	0.34	2370	–	–	–	0.86	0.35	938
Household income per month (in CHF 10)	371	183.94	2822	371.35	184.60	2012	–	–	–	370.40	182.40	810
Education (years)	12.58	2.27	3312	12.61	2.29	2383	–	–	–	12.50	2.22	929
Swiss passport (yes = 1)	0.92	0.28	3297	0.91	0.28	2354	–	–	–	0.92	0.26	943
Parent (yes = 1)	0.66	0.47	3349	0.66	–	2403	–	–	–	0.67	0.47	946
Residence duration (years)	31.44	20.4	3301	31.20	20.50	2368	–	–	–	32	20.3	933
Environmental concern	2.95	0.57	3300	2.95	0.57	2367	–	–	–	2.94	0.57	933

The number of observations (*n*) in the samples corresponds to the sample before multiple imputations. After multiple imputations, the number of observations (*n*) including imputed cases in the OLS models is 3005 (see Tables 3–5)

In addition, we controlled for parenthood, assuming that individuals choose their place of residence based on their family life and assuming that parenthood influences individuals’ resource use. We also controlled for participants’ period of residence, as we assumed that the period of residence would be related to individuals’ life satisfaction (possibly due to a selection effect).

Furthermore, we used respondents’ level of environmental concern as an explanatory variable (Table 2). Environmental concern was measured by a set of items capturing the affective, cognitive, and conative dimensions of environmental concern, as suggested by Diekmann and Preisendörfer (2001). In our case, six items (e.g. “Politics in our country does far too little for environmental protection”) were combined into one index (*M* = 2.9, Cronbach’s *α* = 0.80, *n* = 3300). Higher values indicate higher environmental concern.

### Dealing with Missing Cases

Since the variable household income contained 536 missing values (15.96%) and our vignette-based indicator of resource use contained 576 missing values (17.15%, not including the 142 we excluded from the outset), we conducted multiple imputations using the statistics software Stata (Allison, 2001). After excluding cases with missing values in categorical variables, such as the index of the municipality in which the respondent lived, we used the multivariate normal model for data augmentation and included all variables used in the final analytical models: that is, the dependent and independent variables and all control variables. We imputed 30 (*m*) datasets. Higher imputations were no longer able to increase relative efficiency (*RE* = 0.99). In order to test the robustness of our results based on imputed values, we also fitted OLS regression models without imputations. These additional analyses are reported in Appendix B. We found no substantial differences between the results with and without multiple imputations.

### Data Analysis Strategy

We tested our hypotheses regarding the association between resource use and general life satisfaction and its functional form using OLS regression models with cluster-robust standard errors, accounting for clustering at the municipality level. We used *α* = 5% as the cut-off for statistical significance for two-sided tests. First, we conducted a simple OLS regression to obtain the relationship between resource use and life satisfaction, which is postulated as a positive relationship in Hypothesis 1a. To test whether the positive relationship increases at a decreasing rate, as postulated in Hypothesis 1b, we compared multiple OLS regression models including control variables with and without log-transformed independent variables. To test the difference between the RNPs and the control regions, as postulated in Hypothesis 2a, we included a binary variable that distinguished between park and non-park regions (0 = control region, 1 = park) as an independent variable. We used one model to test the difference in life satisfaction and another model to test the difference in resource use between the RNPs and the control regions. To test whether the relationship between resource use and life satisfaction was weaker for park inhabitants than for individuals living outside the RNPs (Hypothesis 2b), we tested the interaction between these variables in another model by multiplying the park/non-park dummy with the resource use variable (park/non-park × resource use). To identify explanations for the results regarding our hypotheses, we included environmental awareness as an independent variable. To test Hypotheses 3a and 3b, we used the categorical variable indicating the region (the UBE, GNP, JPA, or control regions) and tested its interaction with the resource use variable, respectively. In addition, we fitted models with factors computed by an EFA to test the relations between specific areas of satisfaction, such as infrastructure and personal financial situation, and resource use. These additional analyses are reported in Appendix A.

## Results

Table 3 reports the results of four OLS regression models examining the relationship between individuals’ resource use and life satisfaction. The result of the simple OLS regression (M1) shows that, contrary to Hypothesis 1a, the relationship is negative (*b* = − 0.032, *p* < 0.001). This result does not change substantially when multiple regression is used (M2). A 10-point increase in the vignette-based measure of resource use (about half the scale) decreases the index of life satisfaction by 0.32 points. Although statistically significant, this coefficient evidences a substantially weak relationship. The additional analyses with OLS models using satisfaction with infrastructure, satisfaction with work and financial matters, and the global index of satisfaction as outcome variables support this finding (see Appendix A). These results led us to reject Hypothesis 1a.

**Table 3:** OLS regression models of life satisfaction with and without log transformation of the resource use variable

	Life satisfaction							
	M1		M2		M3 (log.)		M4 (log.)	
	Coef	SE	Coef	SE	Coef	SE	Coef	SE
Resource use (1–21)	− 0.032***	0.007	− 0.032***	0.007	− 0.287***	0.061	− 0.277***	0.065
Age (years)			− 0.012	0.008			− 0.012	0.008
Age × age			0.000**	0.00008			0.000*	0.000
Gender (female = 1)			0.116**	0.048			0.115*	0.048
Single household (no = 1)			0.368**	0.128			0.365**	0.128
Household income per month (in CHF 10)			0.001	0.001			0.001	0.001
Education (years)			0.005	0.012			0.005	0.012
Swiss passport (yes = 1)			0.075	0.098			0.079	0.098
Parent (yes = 1)			0.119	0.074			0.116	0.074
Residence duration (years)			0.001	0.002			0.001	0.002
Environmental concern			− 0.128*	0.059			− 0.125*	0.059
Constant	8.699***	0.067	8.386***	0.259	9.021***	0.132	8.670***	0.415
Number of observations	3005		3005		3005		3005	
Number of clusters	54		54		54		54	
Adjusted *R*^2^	0.007		0.037		0.008		0.037	

The table lists coefficient estimates and cluster-robust standard errors (***p < 0.001, **p < 0.01, *p < 0.05) for two-sided tests of simple and multiple OLS regression models with multiple imputations of missing values. The outcome variable is life satisfaction. In models 3 and 4 the satisfaction variable is log-transformed. The income variable of all four models is resource use. The number of clusters corresponds to the number of municipalities

The rejection of Hypothesis 1a made testing Hypothesis 1b obsolete. Instead, and because the relationship turned out to be negative, we tested whether the relationship between resource use and life satisfaction decreases at a decreasing rate. Since the log transformation of the resource use variable (M3 and M4) does not substantially improve model goodness of fit, we did not find support for this ad hoc hypothesis either (adj. *R*^2^ of M1 = 0.007 and adj. *R*^2^ of M3 = 0.008, adj. *R*^2^ of M2 = 0.037 and adj. *R*^2^ of M4 = 0.037).

The results for most of the control variables included in the multiple regression models M2 and M4 in Table 3 are in line with our expectations, as derived from other studies and theories. Female respondents were more satisfied with their lives than male respondents, younger and older people were more satisfied with their lives than middle-aged people (the negative coefficient of age and the positive coefficient of age squared indicates a u-shaped relation between age and life satisfaction), and people living in collective households were more satisfied with their lives than people living in single households. Environmental concern shows a negative relationship with life satisfaction. However, contrary to previous studies, we found no evidence that nationals (owning a Swiss passport) are more satisfied than foreigners or that education, parenthood, household income, or the residence duration are related to life satisfaction.

Table 4 reports the results of the OLS regressions testing whether the resource use of people living in RNPs is smaller and their life satisfaction higher than those of people living in comparable non-park regions, as postulated in Hypothesis 2a. The multiple regression model M5 shows an insignificant relationship between regions (park and non-park) and life satisfaction. The relation between regions and resource use shown in model M7 is also insignificant. This finding is corroborated by the additional analyses of the relationships between regions and satisfaction with infrastructure, satisfaction with work and financial matters, and the global index of satisfaction. The effects are not significant for these alternative operationalizations of satisfaction either (see Appendix A). These results indicate that the resource use of individuals in RNPs is not lower and that their life satisfaction is not higher than in the control group. This leads us to reject Hypothesis 2a.

**Table 4:** OLS regression models for resource use and life satisfaction and park and non-park regions, including park/non-park x resource use as an interaction term

	Life satisfaction				Resource use	
	M5		M6		M7	
	Coef	SE	Coef	SE	Coef	SE
Resource use (1–21)			− 0.031	0.015		
Lives in park (yes = 1)	0.057	0.059	0.074	0.185	0.145	0.200
Resource use × lives in park			− 0.001	0.017		
Age (years)	− 0.010	0.008	− 0.012	0.008	− 0.063*	0.028
Age × age	0.000**	0.000	0.000**	0.000	0.001	0.000
Gender (female = 1)	0.128 **	0.049	0.116**	0.048	− 0.358*	0.136
Single household (no = 1)	0.370**	0.129	0.367***	0.128	− 0.090	0.266
Household income per month						
(in CHF 10)	0.002	0.001	0.001	0.001	− 0.008**	0.003
Education (years)	0.007	0.012	0.004	0.012	− 0.071	0.038
Swiss passport (yes = 1)	0.099	0.101	0.077	0.098	− 0.673*	0.274
Parent (yes = 1)	0.129	0.073	0.119	0.074	− 0.303	0.205
Residence duration (years)	0.001	0.002	0.001	0.002	0.005	0.005
Environmental concern	− 0.088	0.058	− 0.128*	0.058	− 1.239***	0.146
Constant	7.770***	0.365	8.337***	0.419	18.041***	0.848
Number of observations	3005		3005		3005	
Number of clusters	54		54		54	
Adjusted *R*^2^	0.030		0.036		0.071	

The table lists coefficient estimates and cluster-robust standard errors (***p < 0.001, **p < 0.01, *p < 0.05) for two-sided tests of simple and multiple OLS regression models with multiple imputations of missing values. The outcome variable of M5 and M6 is life satisfaction. The outcome variable of M7 is resource use. The income variable is the region (park and non-park). M6 includes the interaction term of resource use and respondents living in RNPs (= 1) or in the control regions (= 0). The number of clusters corresponds to the number of municipalities

Hypothesis 2b suggested a weaker positive relationship between resource use and life satisfaction for park inhabitants than for individuals living outside RNPs. Given that we found a negative relationship between the two variables (Table 3), we tested whether this negative relationship was stronger for park inhabitants than for the control group. M6 in Table 4 presents the results of the interaction model. The interaction term (park/non-park × resource use) is not significant, meaning that there is no support for our hypothesis of either a weaker positive or a stronger negative relationship between resource use and life satisfaction for park inhabitants as compared to individuals living outside RNPs.^2^

Table 5 provides the results of the OLS regressions testing whether the resource use of individuals in the UBE is lower and their life satisfaction higher than those in the GNP and JPA (Hypothesis 3a). The results of M8 indicate a significant difference in resource use between the UBE and the two other RNPs. On the 21-point scale of our resource use variable, the JPA scores 0.634 points lower and the GNP scores 0.639 points lower than the UBE.

**Table 5:** OLS regression models for resource use and life satisfaction, including the three regional nature parks (and the control group) x resource use as an interaction term, clustered by municipalities

	Resource use		Life satisfaction					
	M8		M9		M10		M11	
	Coef	SE	Coef	SE	Coef	SE	Coef	SE
Resource use (1–21)					− 0.018	0.011	− 0.020	0.011
UBE (ref. cat.)								
JPA	− 0.634*	0.257	− 0.219**	0.064	0.086	0.173	− 0.007	0.169
GNP	− 0.639*	0.297	− 0.082	0.059	0.133	0.136	0.060	0.132
Control group	− 0.560*	0.236	− 0.151*	0.070	− 0.030	0.190	− 0.077	0.200
*Resource use variable x the four regions*								
JPA					− 0.031	0.017	− 0.026	0.017
GNP					− 0.022	0.014	− 0.016	0.014
Control group					− 0.013	0.018	− 0.010	0.019
Age (years)							− 0.011	0.00008
Age x age							0.000*	0.000
Gender (female = 1)							0.110*	0.048
Single household (no = 1)							0.373	0.127
Household income per month (in CHF 10)							0.002	0.001
Education (years)							0.007	0.012
Swiss passport (yes = 1)							0.052	0.094
Parent (yes = 1)							0.116	0.071
Residence duration (years)							0.000	0.002
Environmental concern							− 0.116	0.059
Constant	10.915***	0.166	8.478***	0.052	8.671***	0.099	8.305***	0.381
Number of observations	3005		3005		3005		3005	
Number of clusters	54		54		54		54	
Adjusted *R*^2^	0.004		0.002		0.010		0.041	

The table lists coefficient estimates and cluster-robust standard errors (***p < 0.001, **p < 0.01, *p < 0.05) for two-sided tests of simple and multiple OLS regression models with multiple imputations of missing values. The outcome variable of M8 is the resource use indicator. The outcome variable of M9 to M11 is life satisfaction. The income variable of M8 and 9 represents the regions. M10 and 11 include resource use as the income variable and the interaction term of resource use and the four regions. The number of clusters corresponds to the number of municipalities

Model M9 indicates a significant difference between the UBE and the JPA in terms of life satisfaction. On the 11-point scale of the satisfaction variable, the JPA scores 0.219 points lower than the UBE. We found no evidence for a difference in life satisfaction between the UBE and the GNP. Based on these results, we reject Hypothesis 3a: although life satisfaction is higher in the UBE than in the JPA, so is resource use.

Hypothesis 3b suggests that the strength of the positive correlation between resource use and life satisfaction would be moderated by the age of the RNPs. The two models with interaction terms included (M10, M11) do not substantially differ in terms of effects or significance (with and without control variables). In both models, the interaction terms are not significant. Thus, the strength of the relationship between resource use and life satisfaction is not moderated by the age of the RNPs, and Hypothesis 3b must be rejected.

## Discussion and Conclusion

The aim of this study was to investigate whether RNP status affects resource use, life satisfaction, and the relationship between the resource use and life satisfaction of RNP inhabitants. Also, the moderating effect of the age of RNPs on the relationship between resource use and life satisfaction was examined. Contrary to existing literature showing that the establishment of protected areas is positively related to the well-being of the inhabitants of these areas (Bonet-García et al., 2015), we find no significant differences in resource use or life satisfaction between people living in RNPs and people living in comparable, non-park regions—either overall or for the three investigated RNPs separately. Moreover, contrary to theoretical arguments (Schor, 2001; Veblen, 1973) and empirical evidence (Lenzen & Cummins, 2013; Wang et al., 2015) suggesting a positive relationship between resource use and life satisfaction, our results indicate a statistically significant, albeit substantially small, negative relationship between resource use and life satisfaction. We also do not find support for the hypothesis that the relationship between resource use and life satisfaction is moderated by the age of RNPs. Although people living in the oldest RNP (the UBE) score higher on life satisfaction, their resource use is also higher on average.

What conclusions can we draw given that we did not find the expected differences in resource use and life satisfaction between RNPs and control regions? On the one hand, one interpretation of our results can be that the activities of the RNPs have an effect on resource use and life satisfaction beyond the parks’ borders. The nearby control regions might benefit from the RNPs' activities, and therefore the two types of areas will not significantly differ in regard to the relation between resource use and life satisfaction. On﻿ the other hand, the insignificant result can be interpreted as suggesting that the activities of the RNPs are not sufficiently effective to affect individuals’ lives to a greater extent than those in other areas, or else that these activities are not of the kind that are capable of affecting individuals’ lives. Here it is worth observing that important factors relating to individuals' life satisfaction, such as social relations and equality (e.g. Wiesli et al., 2021), might not be influenced by RNPs, since these factors are not a specific target of RNP activities (Federal Office for the Environment, 2019), which mostly focus on nature and landscape conservation. Moreover, the activities that are carried out in RNPs are restricted by the limited financial and staff resources of RNP management.

It might be expected that the RNPs’ activities regarding environmental education should influence park inhabitants' intentions and behavior so as to induce them to use fewer resources than individuals living in the control regions, as we argued. However, our insignificant result seems not to confirm this hypothesis. In seeking to understand our result, it is worth bearing in mind that although environmental education and knowledge are important prerequisites for resource-saving behavior, empirical studies have repeatedly found that environmental education and knowledge do not necessarily affect resource-saving behavior (Kollmuss & Agyeman, 2002; Liobikienė & Poškus, 2019; Tofighi & Jackson, 2022). People tend to focus on behavior (i.e. sorting waste) that has a relatively low impact on resource-saving (Moser & Kleinhückelkotten, 2018). To lead individuals to lower their resources use in an effective and ecologically beneficial way it is important for environmental education to enable individuals to get knowledge about the ecologically relevant life areas and the behaviour that reduces their resource use in an efficient way. Importantly, government regulations and incentives, as well as infrastructure—for example, renewable energies or public transport—should elicit this ecologically beneficial behavior (Brand & Wissen, 2021). In regard to RNPs, such regulations and incentives are beyond their remit, as they require political and legal processes. Thus, although RNPs can initiate infrastructure projects, such as hiking or cycling routes, and can indirectly influence the development of sustainable infrastructure (for example, by their advice to and co-work with responsible bodies such as municipalities), they have so far limited possibilities to directly provide infrastructure.

What can we conclude from the finding that resource use and life satisfaction are weakly negatively related? According to Inglehart, individuals' pursuit of materialistic values decreases as a society becomes more prosperous (Inglehart, 2015). In Switzerland, both average life satisfaction and household income per capita are above the OECD average (OECD, 2020). In our studied regions, social status might therefore no longer be achieved through obtaining material goods (Veblen, 1973), and individuals may thus no longer strive extensively to obtain material goods (Inglehart, 2015). Excessive consumption might even be associated with negative values in certain societies and lead to social disapproval—also due to people’s increasing awareness of the negative impacts such consumption has on the environment and climate. Rejection by social peers might decrease individuals’ satisfaction.

Our study does not show whether there is a causal relation going from higher satisfaction to lower resource use. However, assuming that the negative relation between resource use and life satisfaction is due to a decrease in materialistic values and the social disapproval of excessive consumption, the maintenance of a high level of life satisfaction would achieve a double social gain. For policy- and decision-makers, this means that efforts to maintain individuals’ life satisfaction at a high level by means of non-material qualities could further reduce individuals’ desire for consumption and resource use.

A further explanation for the negative relationship between resource use and life satisfaction could be that people with higher incomes also spend much of their time working. Several findings indicate that too high a workload, and associated work pressure, can reduce life satisfaction (e.g. Amagasa & Nakayama, 2013; Hsu et al., 2019; Zadow et al., 2021). Moreover, many empirical studies suggest that high income is linked to high resource use (e.g. Bruderer Enzler & Diekmann, 2019). Thus, an interpretation of our result could be that working less decreases resource use and increases life satisfaction. However, the discussion on whether part-time work, as compared to full-time work, significantly leads to higher life satisfaction is controversial (e.g. Logan et al., 1973; Montero & Rau, 2015).

Our study has some limitations. Due to the scope of the survey, we cannot compare our measures of resource use and life satisfaction with other regions of Switzerland that are further away from the selected RNPs. Moreover, our selection of control groups in non-park regions was restricted by the criterion of being neither too different nor too similar to the park regions. The geographical proximity made it harder to find significant differences in the relationship between resource use and life satisfaction across the regions we studied. Future surveys should compare resource use and life satisfaction in park regions with the averages of statistically similar populations in regions of Switzerland that are located further away from the selected park regions. Such a comparison would allow us to ascertain whether the first (positive) or the second (negative) explanation for our findings is more plausible. If, in regions located further away, the average resource use is higher and the average life satisfaction lower, it would mean that our null finding results from a spillover effect. If the averages are the same, or if resource use is higher and life satisfaction lower in the RNPs, it would instead mean that our null finding provides evidence that RNP activities have no effect on the relationship between resource use and life satisfaction.

Further studies, going beyond the scope of the present study, could, for example, compare urban and rural areas and give insights into social or cultural differences that might explain the negative relation between life satisfaction and resource use. Moreover, future research could address the role of individuals’ participation in the RNPs and their effects on resource use and life satisfaction. Research has shown that community participation and the resulting identification with sustainable development and nature protection in, for example, UNESCO Biospheres is crucial for promoting sustainable development and nature protection (Berghöfer & Berghöfer, 2006; Cohen-Shacham et al., 2019; Jordan & Adger, 2009; Stoll-Kleemann & Welp, 2008; Stoll-Kleemann et al., 2010). The Swiss bottom-up approach to establishing an RNP entails a different starting position with regard to participation from the very beginning and might have a different effect on the relationship between resource use and life satisfaction than the establishment of nature parks in other countries, which do not result from a direct democratic process. A comparison of RNPs and parks in other countries with other establishment processes could elicit new insights into the effects of democracy on the relationship between resource use and life satisfaction. Moreover, investigations into the effects of different types of participation in the activities of the RNP might provide insights into the way RNPs can engage individuals in their activities to help manage their resource use and life satisfaction.

## Supplementary Information

Below is the link to the electronic supplementary material.

Supplementary file1 (DOCX 58 KB)

## Data Availability

Available upon request.

## Footnotes

1 The complete WWF calculation includes carbon emissions from non-food consumption, services, cruises, construction of houses, and the relative proportions of seasonal and non-seasonal food.

2 The *p*-value of the coefficient of resource use in M6 is insignificant (*b* = −0.031, *p* = 0.052). This can be explained by the interaction term, which drains the power of the model. If the interaction term is removed, the coefficient is significantly negative (*b* = −0.032, *p* < 0.001), as in model M2, for example.

## Code availability

Available upon request.
