# Supplementary material for: Does Living in a Protected Area Reduce Resource Use and Promote Life Satisfaction? Survey Results from and Around Three Regional Nature Parks in Switzerland
Source: Soc Indic Res. 2023 Jul 7;169(1-2):341–64. doi: 10.1007/s11205-023-03164-z (PMC10462571; doi:10.1007/s11205-023-03164-z)
Supplement: Supplementary file 1 — Supplementary file1 (DOCX 58 KB) [file 11205_2023_3164_MOESM1_ESM.pdf]

Appendix to the article "Does Living in a Protected Area Reduce Resource Use and Promote Life Satisfaction? Survey Results from and Around Three Regional Nature Parks in Switzerland"

Thea Xenia Wiesli[1]*, Wojtek Przepiorka[2]

[1] University of Bern, Centre for Development and Environment, Bern, Switzerland, thea.wiesli@unibe.ch, ORCID-ID: 0000-0002-3610-3058

[2] Utrecht University, Department of Sociology, Utrecht, Netherlands, w.przepiorka@uu.nl, ORCID-ID: 0000-0001-9432-8696

* Correspondence: thea.wiesli@unibe.ch

Appendix A: OLS regression with factors and global index of satisfaction

In order to provide more specific information and validate our results, we tested in additional analyses whether the relation between life satisfaction and resource use is qualitatively different if we use specific factors of life satisfaction and a global satisfaction index instead of the variable on general life satisfaction (see the "Results" section in the article). We ran factor analyses on 21 variables about people's satisfaction and called one of the two resulting factors "satisfaction with infrastructure". This includes the variables satisfaction with leisure offers, satisfaction with footpaths, satisfaction with cycle routes, satisfaction with road infrastructure, satisfaction with public transport, and satisfaction with basic supplies, such as streets, electricity, water etc. ($\chi^2(210)$ = 8129.81, $p < 0.001$; KMO = 0.870; 2.52% of variance explained, n = 1,644). The other resulting factor is called "satisfaction with work and financial matters" and includes the variables satisfaction with one's financial situation, satisfaction with employment, and satisfaction with rent costs ($\chi^2(45)$ = 3835.44, $p < 0.001$; KMO = 0.831; 2.20% of variance explained, n = 1,680). Taking these two factors as outcome variables, we ran OLS regression models with multiple imputations (see Tables S1 and S2).

The models including the factor "satisfaction with infrastructure" as the outcome variable indicate a significantly negative relation between satisfaction with infrastructure and resource use (Table S1), as does the model presented in the article that uses the global satisfaction index as the outcome variable (see Table 3). The coefficients in models M12 and M13 in Table S1 do not substantially differ from the ones in the article (Table 3; M1: b = -0.032, $p < 0.001$, M2: b = -0.032, $p < 0.001$).

Table S1
OLS regression models for satisfaction with infrastructure and resource use, clustered by municipalities

	Satisfaction with infrastructure			
	M12		M13	
	Coef.	SE	Coef.	SE
Resource use (1–21)	-0.013***	0.003	-0.006**	0.002
Age (years)			0.045***	0.003
Age x age			-0.001***	0.000
Gender (female = 1)			-0.082***	0.016
Single household (no = 1)			-0.049*	0.022
Household income per month (in CHF 10)			0.001***	0.000
Education (years)			0.018***	0.004
Swiss passport (yes = 1)			0.017	0.033
Parent (yes = 1)			0.061*	0.024
Residence duration (years)			0.001*	0.000
Environmental concern			0.021	0.017
Constant	0.645***	0.028	-0.594***	0.095
Number of observations	3,005		3,005	
Number of clusters	54		54	
Adjusted R^2	0.008		0.252	

Notes: The table lists coefficient estimates and cluster-robust standard errors (*** $p < 0.001$, ** $p < 0.01$, * $p < 0.05$, for two-sided tests) of simple and multiple OLS regression models with multiple imputations of missing values. The outcome variable of models 12 and 13 is a factor of satisfaction, namely satisfaction with infrastructure. The income variable represents individuals' resource use. The number of clusters corresponds to the number of municipalities.

The simple model including the factor "satisfaction with work and financial matters" as the outcome variable likewise indicates a significant negative relation (Table S2). The coefficients in the models M14 and M15 in Table S2 are only slightly lower than those in the models using the life satisfaction variable presented in the article (Table 3, M1: $b = -0.032, p < 0.001$, M2: $b = -0.032, p < 0.001$). Both models indicate a significant negative relation between satisfaction with infrastructure and resource use, as does the model using the global satisfaction index presented in the article (see Table 3).

Table S2
OLS regression models for satisfaction with work and financial matters and resource use, clustered by municipalities

	Satisfaction with work and financial matters			
	M14		M15	
	Coef.	SE	Coef.	SE
Resource use (1–21)	-0.014***	0.003	-0.007**	0.002
Age (years)			0.046***	0.003
Age x age			-0.001***	0.000
Gender (female = 1)			-0.073***	0.016
Single household (no = 1)			-0.057*	0.022
Household income per month (in CHF 10)			0.002***	0.000
Education (years)			0.017***	0.004
Swiss passport (yes = 1)			0.006	0.033
Parent (yes = 1)			0.065**	0.024
Residence duration (years)			0.001*	0.000
Environmental concern			0.020	0.016
Constant	0.664	0.028	-0.572***	0.090
Number of observations	3,005		3,005	
Number of clusters	54		54	
Adjusted R^2	0.009		0.260	

Notes: The table lists coefficient estimates and standard errors (*** $p < 0.001$, ** $p < 0.01$, * $p < 0.05$) of a simple and multiple OLS regression model with multiple imputations of missing values. The outcome variable in models 14 and 15 is a factor of satisfaction, namely satisfaction with work and financial matters. The income variable represents individuals' resource use. The number of clusters corresponds to the number of municipalities.

In the "Results" section of the article, we present the results of models testing whether people living in parks have lower resource use and higher self-reported life satisfaction than people living in comparable, non-park regions (HP 2a). Here we tested the same hypothesis with the factors "satisfaction with infrastructure" and "satisfaction with work and financial matters" as outcome variables. Like the models reported in the article (Table 4; M5: $b = 0.057, p > 0.5$, M7: $b = 0.145, p > 0.5$) the models M16 and M17 in Table S3 indicate an insignificant relation between the two regions (park and non-park) and the two factors. Estimating the models with "satisfaction with infrastructure" and "satisfaction with work and financial matters" thus does not provide support for our hypothesis either. Levels of satisfaction with these specific aspects (infrastructure, work, and financial situation) are not higher for park inhabitants than for the control group.

Table S3
OLS regression models for satisfaction with infrastructure, satisfaction with work and financial matters and park and non-park regions, clustered by municipalities

	Satisfaction with infrastructure		Satisfaction with work and financial matters	
	M16		M17	
	Coef.	SE	Coef.	SE
Lives in park (yes = 1)	-0.006	0.019	-0.007	0.019
Age (years)	0.046	0.003	0.046***	0.003
Age x age	-0.001***	0.000	-0.001***	0.000
Gender (female = 1)	-0.079***	0.015	-0.070***	0.016
Single household (no = 1)	-0.049***	0.022	-0.057*	0.022
Household income per month (in CHF 10)	0.001***	0.000	0.002***	0.000
Education (years)	0.019***	0.004	0.018***	0.004
Swiss passport (yes = 1)	0.021	0.033	0.011	0.033
Parent (yes = 1)	0.063***	0.024	0.067**	0.024
Residence duration (years)	0.001	0.000	0.001*	0.000
Environmental concern	0.029	0.017	0.029	0.016
Constant	-0.699***	0.085	-0.689***	0.080
Number of observations	3,005		3,005	
Number of clusters	54		54	
Adjusted R^2	0.250		0.258	

Notes: The table lists coefficient estimates and cluster-robust standard errors (***$p < 0.001$, **$p < 0.01$, *$p < 0.05$, for two-sided tests) of simple and multiple OLS regression models with multiple imputations of missing values. The outcome variables in models 16 and 17 are factors of satisfaction. The income variable represents individuals living either in RNPs or in the control group. The number of clusters corresponds to the number of municipalities.

To test the validation of the life satisfaction variable in the main analyses of the article (see Table 2) and the factors above, we also created a global index of all of the 21 variables relating to people's life satisfaction. Here we tested in an additional analysis whether the relation between satisfaction and resource use is qualitatively different if we use the global satisfaction index (see Table S4) instead of the variable general life satisfaction, as in the model of the article. The models (18 and 19) in Table S4 including the global index "satisfaction" as the outcome variable indicate a significantly negative relation between satisfaction with resource use, as does the model presented in the article that includes the single variable of general life satisfaction as the outcome variable (Table 3; M1: $b = -0.032, p < 0.001$, M2: $b = -0.032, p < 0.001$).

Table S4
OLS regression models for the global index of life satisfaction and resource use, clustered by municipalities

	Life satisfaction (index)			
	M18		M19	
	Coef.	SE	Coef.	SE
Resource use (1–21)	-0.021***	0.005	-0.024***	0.005
Age (years)			-0.018**	0.005
Age x age			0.000***	0.000
Gender (female = 1)			0.134***	0.030
Single household (no = 1)			0.006	0.068
Household income per month (in CHF 10)			0.002**	0.001
Education (years)			-0.013	0.009
Swiss passport (yes=1)			0.287**	0.082
Parent (yes = 1)			0.052	0.039
Residence duration (years)			-0.116**	0.001
Environmental concern			8.345***	0.037
Constant	8.234***	0.051	0.005***	0.259
Number of observations	3,005		3,005	
Number of clusters	54		54	
Adjusted R^2	0.006		0.057	

Notes: The table lists coefficient estimates and cluster-robust standard errors (*** p < 0.001, ** p < 0.01, * p < 0.05) for two-sided tests of simple and multiple OLS regression models with multiple imputations of missing values. The outcome variable of models 18 and 19 is the global index of satisfaction and the income variable is the indicator of resource use.

In the "Results" section of the article, we present models testing whether people living in parks have lower resource use and higher self-reported satisfaction than people living in comparable, non-park regions (HP 2a). Here we tested the same hypothesis with the global index of satisfaction. Like the models reported in the article (Table 4; M5: $b = 0.057, p > 0.5$, M7: $b = 0.145, p > 0.5$), the models M20 and M21 in Table S5 indicate an insignificant relation between the two regions (park and non-park) and the index of satisfaction. Thus, estimating the models with the index does not provide support for our hypothesis either.

Table S5
OLS regression models for global index of satisfaction and park and non-park regions, clustered by municipalities

	Life satisfaction (index)			
	M20		M21	
	Coef.	SE	Coef.	SE
Lives in park (yes = 1)	-0.010	0.066	0.004	0.055
Age (years)			-0.016**	0.005
Age x age			0.000***	0.000
Gender (female = 1)			0.143 ***	0.029
Single household (no = 1)			0.008	0.069
Household income per month (in CHF 10)			0.002**	0.001
Education (years)			-0.011	0.009
Swiss passport (yes = 1)			0.303**	0.083
Parent (yes = 1)			0.060	0.039
Residence duration (years)			0.005**	0.001
Environmental concern			-0.087*	0.038
Constant	8.005***	0.059	7.917***	0.237
Number of observations	3,005		3,005	
Number of clusters	54		54	
Adjusted R^2	-0.0003		0.049	

Notes: The table lists coefficient estimates and cluster-robust standard errors (***$p < 0.001$, **$p < 0.01$, *$p < 0.05$, for two-sided tests) of simple and multiple OLS regression models with multiple imputations of missing values. The outcome variable in models 20 and 21 is a global index of life satisfaction. The income variable represents individuals living either in RNPs or in the control group. The number of clusters corresponds to the number of municipalities.

Appendix B: OLS regression without multiple imputations

In the estimations used in our article, we applied multiple imputations due to a large number of missing values in one of the control variables. However, multiple imputations provide a level of uncertainty in regard to the imputed values. Therefore, we additionally tested here the most important hypotheses, 1a and 2a, without imputations, to see whether these models showed any significant differences compared to the models with imputations.

In order to test Hypothesis 1a – the relation of people's self-reported satisfaction with their resource use – we ran the same simple and multiple models (M22 and M23 in Table S6) as are described in the "Results" section of the article (M1 and M2 in Table 3), but without multiple imputations. The results with and without multiple imputations do not differ substantially. The models without imputations indicate a significant negative relation between resource use and life satisfaction, as do the models with multiple imputations (M1 in Table 3: $b = -0.032$, $p < 0.001$). The same applies to the coefficients of the multiple regression models (M2 in Table 3: $b = -0.032$, $p < 0.001$).

Table S6

OLS regression models for resource use and life satisfaction, clustered by municipalities

	Life satisfaction			
	M22		M23	
	Coef.	SE	Coef.	SE
Resource use (1–21)	-0.031***	0.008	-0.030**	0.009
Age (years)			-0.014	0.010
Age x age			0.000*	0.000
Gender (female = 1)			0.136**	0.048
Single household (no = 1)			0.263	0.134
Household income per month (in CHF 10)			0.001	0.001
Education (years)			-0.009	0.013
Swiss passport (yes = 1)			0.092	0.097
Parent (yes = 1)			0.084	0.076
Residence duration (years)			0.002	0.002
Environmental concern			-0.123	0.063
Constant	8.685***	0.073	8.355***	0.421
Number of observations	2,172		2,172	
Number of clusters	54		54	
R^2	0.007		0.041	

Notes: The table lists coefficient estimates and cluster-robust standard errors (***$p < 0.001$, **$p < 0.01$, *$p < 0.05$, for two-sided tests) of simple and multiple OLS regression models. The outcome variable of models 22 and 23 is the life satisfaction variable (general life satisfaction). The income variable is the resource use indicator. The number of clusters corresponds to the number of municipalities.

Table S7 reports the results of OLS regression models without multiple imputations to test Hypothesis 2a. The hypothesis postulates that people living in parks have lower resource use and higher self-reported satisfaction than people living in comparable, non-park regions. Like in the models with multiple imputations in the article (M5 and M7 in Table 4), the results of the models show insignificant relations between regions (park and non-park) and life satisfaction (see M24 in Table S7) and between regions and resource use (see M25 in Table S7). Thus, the models without multiple imputations show that the resource use of individuals living in parks is not lower, and their life satisfaction is not higher, than the resource use and life satisfaction of those in the control group – just like the models reported in the “Results” section of the article (see Table 4). Accordingly, it can be assumed that the estimations with multiple imputations presented in the article are reliable.

Table S7

OLS regression models for life satisfaction and park and non-park regions, as well as resource use and park and non-park regions, clustered by municipalities

	Life satisfaction		Resource use	
	M24		M25	
	Coef.	SE	Coef.	SE
Lives in park (yes = 1)	0.072	0.053	0.292	0.238
Age (years)	-0.011	0.010	-0.065	0.033
Age x age	0.000	0.000	0.001	0.000
Gender (female = 1)	0.149	0.050	-0.447**	0.157
Single household (no = 1)	0.260**	0.137	0.038	0.283
Household income per month (in CHF 10)	0.002	0.001	-0.008**	0.003
Education (years)	0.010	0.013	-0.062	0.042
Swiss passport (yes = 1)	0.118	0.097	-0.818**	0.281
Parent (yes = 1)	0.097**	0.075	-0.393	0.199
Residence duration (years)	0.002	0.002	0.004	0.005
Environmental concern	-0.082	0.061	-1.332**	0.171
Constant	7.746***	0.394	18.380***	0.911
Number of observations	2,172		2,172	
Number of clusters	54		54	
R^2	0.036		0.086	

Notes: The table lists coefficient estimates and cluster-robust standard errors (*** $p < 0.001$, ** $p < 0.01$, * $p < 0.05$, for two-sided tests) of simple and multiple OLS regression models. The outcome variable of model 24 is satisfaction (life satisfaction in general). The outcome variable of model 25 is the resource use indicator. The income variable represents individuals living either in RNPs or in the control group. The number of clusters corresponds to the number of municipalities.
